# Noninvasive Evaluation of Liver Function in Morbidly Obese Patients

**DOI:** 10.1155/2019/4307462

**Published:** 2019-02-03

**Authors:** Patrick H. Alizai, Isabella Lurje, Andreas Kroh, Sophia Schmitz, Tom Luedde, Julia Andruszkow, Ulf P. Neumann, Florian Ulmer

**Affiliations:** ^1^Department of General, Visceral and Transplantation Surgery, RWTH Aachen University Hospital, Pauwelsstr. 30, 52074 Aachen, Germany; ^2^Department of Gastroenterology, Digestive Diseases and Intensive Care Medicine, RWTH Aachen University Hospital, Pauwelsstr. 30, 52074 Aachen, Germany; ^3^Institute of Pathology, RWTH Aachen University Hospital, Pauwelsstr. 30, 52074 Aachen, Germany; ^4^Department of Surgery, Maastricht University Medical Center, P. Debyelaan 25, 6229 HX Maastricht, Netherlands

## Abstract

**Background:**

More than half of the obese patients develop nonalcoholic fatty liver disease (NAFLD), which may further progress to nonalcoholic steatohepatitis (NASH) and cirrhosis. The aim of this study was to assess alterations in liver function in obese patients with a noninvasive liver function test.

**Methods:**

In a prospective cohort study 102 morbidly obese patients undergoing bariatric surgery were evaluated for their liver function. Liver function capacity was determined by the LiMAx® test (enzymatic capacity of cytochrome P450 1A2). Liver biopsy specimens were obtained intraoperatively and classified according to the NAFLD Activity Score (NAS). NASH clinical score was additionally calculated from laboratory and clinical parameters.

**Results:**

Median liver function capacity was 286 (IQR = 141) *μ*g/kg/h. 27% of patients were histologically categorized as definite NASH, 39% as borderline, and 34% as not NASH. A significant correlation was observed between liver function capacity and NAS (*r* = −0.492; *p* < 0.001). The sensitivity and specificity of the LiMAx® test to distinguish between definite NASH and not NASH were 85.2% and 82.9% (AUROC 0.859), respectively. According to the NASH clinical scoring system, 14% were classified as low risk, 31% as intermediate, 26% as high, and 29% as very high risk. Liver function capacity is also significantly correlated with the NASH clinical scoring system (*r* = −0.411; *p* < 0.001).

**Conclusions:**

Obese patients show a diminished liver function capacity, especially those suffering from type 2 diabetes. The liver function capacity correlates with histological and clinical scoring systems. The LiMAx® test may be a valuable tool for noninvasive screening for NASH in obese patients.

## 1. Introduction

The prevalence of overweight and obesity has dramatically increased in the last decades. Nowadays, more than 600 million adults are obese worldwide [1]. Morbid obesity is accompanied by various other diseases like type 2 diabetes, hypertension, dyslipidemia, and obstructive sleep apnea syndrome [2]. The hepatic manifestation of the metabolic syndrome is the nonalcoholic fatty liver disease (NAFLD) [3]. It includes a wide clinicopathological spectrum ranging from simple steatosis to nonalcoholic steatohepatitis (NASH), which then may progress to liver cirrhosis and hepatocellular carcinoma [4]. The prevalence of NAFLD and NASH in the general population is estimated to be 10-30% and 3-5%, respectively [5]. Among obese people, the prevalence of NAFLD and thus NASH is considerably higher, ranging from 50-90% and 10-50%, respectively [5, 6]. Therefore, screening of patients at risk for developing NASH appears reasonable.

Unfortunately, liver enzymes can be in the normal range in NASH patients, and ultrasound has technical limitations in obese patients. Several noninvasive tests were developed, but at present no marker or scoring system can accurately differentiate NASH from simple steatosis [7]. Therefore, histopathological examination of liver biopsies remains the gold standard for the staging of NAFLD. However, liver biopsy is an invasive technique with well-known risks and is subject to sampling variability [8, 9]. These limitations resulted in a growing interest to find noninvasive approaches for the diagnosis of NASH. In this line, the noninvasive LiMAx® test (Liver Maximum Capacity Test) has already been successfully used in liver surgery, liver transplantation, and bariatric surgery [10–14]. The aim of this study was to evaluate liver function capacity with the LiMAx® test in morbidly obese patients undergoing bariatric surgery.

## 2. Patients and Methods

### 2.1. Study Design

This prospective cohort study was conducted at the interdisciplinary bariatric center of the RWTH Aachen University Hospital between 2013 and 2017. Participants were bariatric surgery candidates with body mass indices of >40 kg/m^2^ or > 35 kg/m^2^ with weight-related comorbidities. Exclusion criteria were age < 18 y, heavy smoking (>15 cigarettes per day), alcohol consumption (>20 g/day), and causes of liver disease other than NAFLD (e.g., viral hepatitis and autoimmune hepatitis). Clinical data (age, body weight, body height, and comorbidities), liver function, and biochemical parameters were recorded preoperatively. Liver biopsies were obtained during bariatric surgery. Data were pseudonymized and saved in a secured database. The study was conducted in accordance with the 1964 Declaration of Helsinki and its later amendments and had received prior approval by the Local Ethics Committee (EK 312/11). Written informed consent was obtained from each patient before enrolment.

### 2.2. Liver Function Capacity

Liver function was measured by the LiMAx® test preoperatively. The LiMAx® test is based on hepatic ^13^C-methacetin (Euriso-top, Saint-Aubin Cedex, France) metabolisms by the cytochrome P450 1A2 system (CYP1A2). A bolus of ^13^C-methacetin (2 mg/kg body weight) was injected intravenously. After injection, ^13^C-methacetin is metabolized into acetaminophen and ^13^CO_2_, which is pulmonary exhaled. The analysis of emerging ^13^CO_2_ was performed by online breath sampling with real-time bedside analysis by a laser-based nondispersive isotope-selective infrared spectroscope (FLIP2, Humedics, Berlin, Germany). The normal range of liver function capacity is considered >315 *μ*g/kg/h [10, 15].

### 2.3. Laboratory Tests

Blood samples were collected preoperatively after an overnight fast. Biochemical parameters were determined at the Institute of Clinical Chemistry of the RWTH Aachen University Hospital. The normal range of alanine aminotransferase (ALT) and aspartate aminotransferase (AST) is 10-50 U/l.

### 2.4. NASH Clinical Scoring System

The NASH clinical scoring system was calculated according to Campos et al. [16]. The scoring system is based on clinical data (hypertension, type 2 diabetes, and sleep apnea syndrome: one point each), race (nonblack: 2 points), and biochemical parameters (AST ≥ 27 U/l and ALT ≥ 27 U/l: one point each). The scoring system was developed to predict NASH particularly in morbidly obese patients.

### 2.5. Liver Histology

A wedge resection on the left lobe of the liver was performed at the beginning of the operation. All histologic specimens were reviewed by a single hepatopathologist (JA), who was blinded to the clinical data. Histopathological analyses were performed according to the NAFLD Activity Score (NAS) by Kleiner et al. [17]. The scoring system comprises different histological features like steatosis, lobular inflammation, hepatocellular ballooning, and fibrosis.

### 2.6. Statistical Analysis

Statistical evaluation was carried out using the SPSS® 24.0 software (SPSS, Chicago, IL, USA). Values are presented as mean and standard deviation or median and interquartile range (IQR) unless otherwise specified. Significance was calculated using the two-sample *t*-test or the Mann-Whitney *U* test in case of normal distributions. A two-sided *p* < 0.05 was considered statistically significant. Correlation was assessed by Pearson's correlation coefficient or Spearman's correlation coefficient. Receiver operating characteristic (ROC) analysis was performed to analyze the sensitivity and specificity of the LiMAx® system.

## 3. Results

### 3.1. Patients' Characteristics

Between 2013 and 2017, a cohort of 102 patients was included in this study. Detailed clinical and demographic data are summarized in Table 1. Seventy of the 102 patients (68.6%) were female. The average age of all patients was 43 ± 11 years. The mean body mass index was 54 ± 9 kg/m^2^ (range: 35-75 kg/m^2^). Obesity-related comorbidities were type 2 diabetes (36.3%), hypertension (63.7%), and obstructive sleep apnea syndrome (49.0%). Gastric bypass was performed in 43 (42.2%) patients and sleeve gastrectomy in 59 (57.8%).

### 3.2. Laboratory Tests and NASH Clinical Scoring System

The mean value for alanine aminotransferase (ALT) was 36.1 ± 26.4 U/l and for aspartate aminotransferase (AST) 27.4 ± 13.3 U/l; both were within the normal range (10-50 U/l). Sixty-six participants (64.7%) had an ALT ≥ 27 U/l and 41 (40.2%) an AST ≥ 27 U/l. These respective patients received one point in the NASH clinical scoring system. Results of the summed points are summarized in Table 1. Fourteen patients (13.7%) were classified as low risk, 32 (31.4%) as intermediate, 26 (25.5%) as high, and 30 (29.4%) as very high risk. The NASH clinical scoring system showed a positive correlation with the NAFLD Activity Score (NAS): *r* = 0.535; *p* < 0.001.

### 3.3. Liver Biopsy

No indication for NASH was observed in 35 specimens (34.3%), 40 were classified as borderline (39.2%), and 27 (26.5%) showed manifest histological signs of NASH. Of the 35 specimens that were classified as no NASH, 25 (71.4%) showed simple steatosis. Median NAS score was 3 (IQR = 3). F1 fibrosis was detected in 47 patients (46.1%), F2 fibrosis in 22 (21.6%), and F3 fibrosis in 10 patients (9.8%). In 23 cases (22.5%), no fibrosis was observed.

### 3.4. Liver Function Capacity

**Figure 1:**
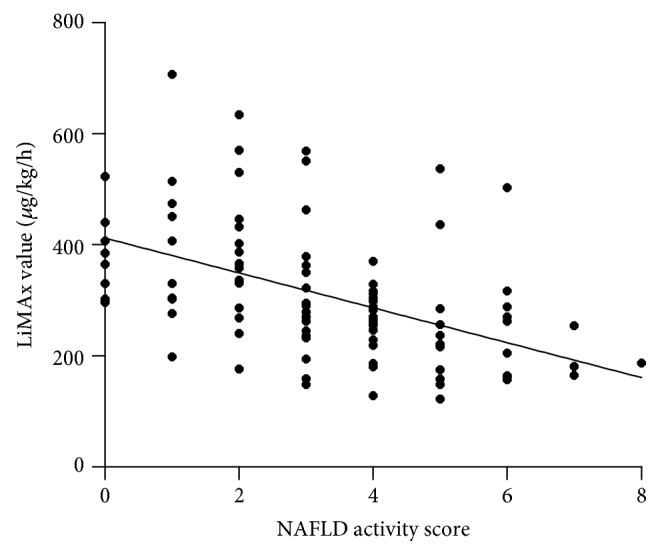
Correlation between NAS score und LiMAx value. NAS score shows a negative correlation to liver function capacity (*r* = −0.492; *p* = 0.001).

**Figure 2:**
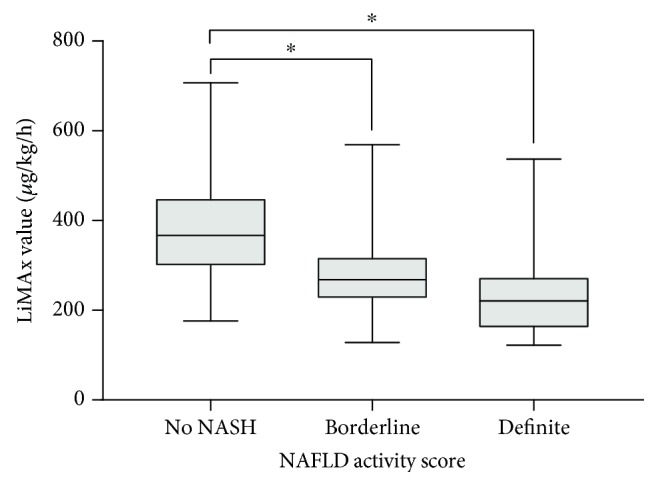
Mean LiMAx value in different NAS groups. Mean LiMAx value in patients without NASH was 388 ± 116 *μ*g/kg/h, which is significantly higher than in patients with borderline (281 ± 93 *μ*g/kg/h; ^∗^*p* < 0.001) or definite NASH (241 ± 104 *μ*g/kg/h; ^∗^*p* < 0.001). No significant difference was found between borderline and definite NASH (*p* = 0.109).

**Figure 3:**
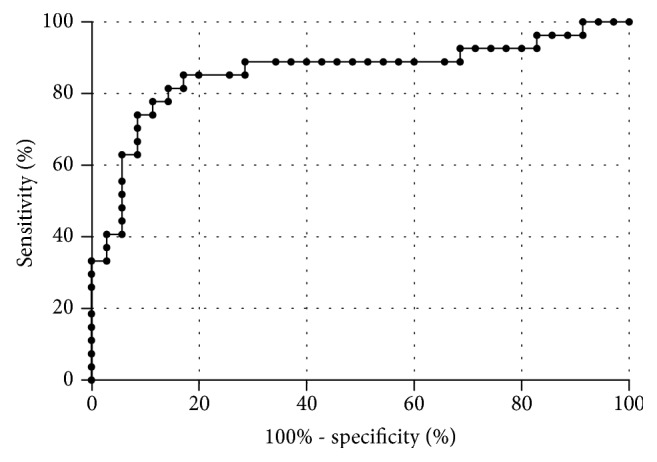
Sensitivity and specificity of LiMAx test. Comparing definite NASH and not NASH, the sensitivity and specificity are 85.2% and 82.9% (AUROC 0.859 and cut-off 288 *μ*g/kg/h), respectively.

Median liver function capacity was 286 (IQR = 141) *μ*g/kg/h (range: 122-707 *μ*g/kg/h). 61.8% patients showed values below the normal range (≤315 *μ*g/kg/h). Liver function capacity negatively correlated with the NAS score (*r* = −0.492; *p* < 0.001) (Figure 1). Mean LiMAx® value in patients without NASH was significantly higher than in patients with borderline NASH (388 vs. 281 *μ*g/kg/h; *p* < 0.001) (Figure 2). The sensitivity and specificity of LiMAx® test to distinguish between definite NASH and not NASH were 85% and 83%, respectively (AUROC 0.859 and cut-off 288 *μ*g/kg/h) (Figure 3).

**Figure 4:**
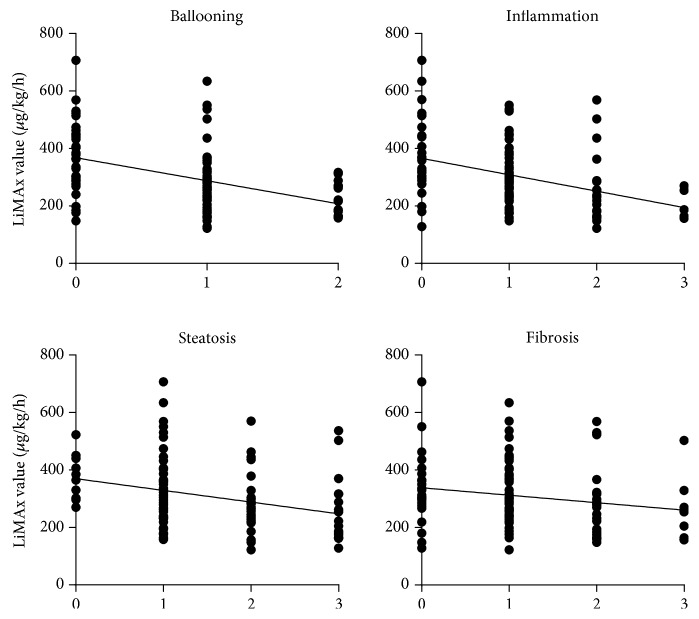
Correlation between different histological features and LiMAx value. Correlations between LiMAx and different histological features were *r* = −0.446 for hepatocellular ballooning (*p* < 0.001), *r* = −0.397 for inflammation (*p* < 0.001), and *r* = −0.305 for steatosis (*p* = 0.002). No correlation was observed between the stage of fibrosis and liver function capacity (*r* = −0.195; *p* = 0.050).

Correlations between LiMAx® and different histological features were: *r* = −0.446 for hepatocellular ballooning (*p* < 0.001), *r* = −0.397 for inflammation (*p* < 0.001), and *r* = −0.305 for steatosis (*p* = 0.002). No correlation was observed between the stage of fibrosis and liver function capacity (*r* = −0.195; *p* = 0.050) (Figure 4).

**Figure 5:**
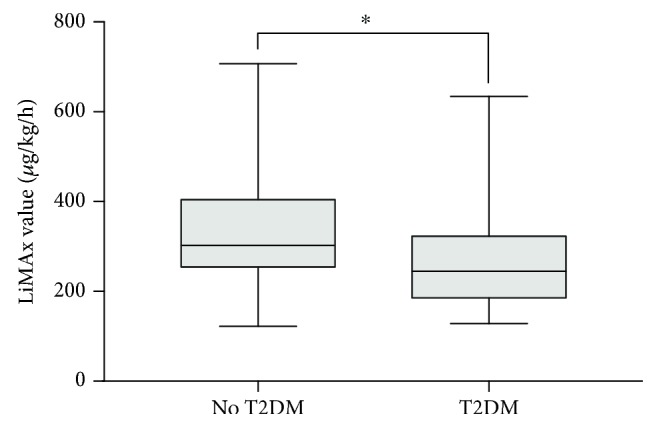
Mean LiMAx value regarding T2 diabetes mellitus. Mean LiMAx value in patients without T2DM (329 ± 121 *μ*g/kg/h) was significantly higher than in patients with T2DM (269 ± 109 *μ*g/kg/h; ^∗^*p* = 0.015).

Liver function capacity negatively correlated with the NASH clinical scoring system (*r* = −0.411; *p* < 0.001). LiMAx® values showed no correlation with preoperative BMI and age. Mean LiMAx® value was significantly lower in patients with T2DM than in patients without T2DM (269 vs. 329 *μ*g/kg/h; *p* = 0.015) (Figure 5).

## 4. Discussion

Nonalcoholic fatty liver disease (NAFLD) is presently the most common liver disorder in the Western world [4], with obesity and insulin resistance playing the major pathophysiological roles in its development [3]. It is therefore not surprising that the prevalence of NALFD among obese patients is alarmingly high. The aim of this study was to assess alterations in liver function capacity in morbidly obese patients.

Median liver function capacity in this bariatric cohort was considerably lower than in normal subjects [10, 15]. Furthermore, liver function capacity correlated to the histological signs of NASH. The LiMAx® value in patients without NASH was significantly higher than in patients with borderline or manifest NASH. The sensitivity and specificity of the test to distinguish between definite NASH and not NASH were 85% and 83%, respectively. With an AUROC value of 0.86, the LiMAx® test method is a promising noninvasive diagnostic tool compared to other noninvasive methods (0.76 to 0.90) [18] and appears to be suitable for the screening of NASH in morbidly obese patients. So far, the test has been applied to assess the pre- and postoperative liver function in liver surgery and liver transplantation [10–12]. The LiMAx® test is based on hepatic ^13^C-methacetin metabolism by the cytochrome P450 1A2 system and can validly determine liver function capacity [10–12, 15]. However, because of the complexity of liver function, different liver function tests are used in practice, such as indocyanine green test (ICG), galactose elimination capacity, and ^99m^Tc-galactosyl serum albumin scintigraphy [19]. Danin et al. evaluated liver function in 26 morbidly obese patients with the ICG test. They reported a correlation between ICG clearance and steatosis but found no correlation between ICG clearance and hepatic inflammation or ballooning [20]. In contrast, LiMAx® values showed a correlation to inflammation and ballooning. This fact seems to be important as hepatic inflammation marks the decisive step from simple steatosis towards steatohepatitis and fibrosis later on [21].

Liver function capacity was significantly lower in patients with T2DM, which underlines the central role of insulin resistance and type 2 diabetes in the pathogenesis and progression of NAFLD [22, 23]. Large population studies have shown that almost all of the NAFLD patients were insulin-resistant [24, 25]. Moreover, elevated liver enzymes in patients with NAFLD are known to be a predictor of T2DM, independently of BMI [26].

Histological examination is certainly the gold standard for diagnosing and staging NAFLD, but unfortunately liver biopsies are not without risk [27]. Liver biopsies are invasive, unpleasant for patients, and technically challenging in obese individuals [24]. A biopsy only represents a very small part of the liver and therefore underlies a sampling variability [9, 24]. Furthermore, the histological definitions of NASH as well as the NAFLD activity score (NAS) are subject to controversy [28, 29]. It has been criticized that the NAS has a wide gray zone (NAS: 3-4) wherein NASH may or may not be present [17, 28].

Because of the high prevalence of NAFLD and the mentioned drawbacks of liver biopsy, much effort is being expended on developing noninvasive diagnostic tools. NAFLD can be detected by magnetic resonance imaging and magnetic resonance spectroscopy [30]. However, the availability is limited, expertise in protocol prescription is needed, and presence of metal implants or claustrophobia has to be considered [18]. Transient elastography or other ultrasound-based tests are more accessible and cheaper, but application can be limited in extremely obese patients [31]. Conventional biochemical parameters are easily obtained, but their sensitivity and specificity to detect NAFLD are low [32]. In the present study, almost all patients had AST and ALT values within the normal range. Several other biochemical markers, such as IL-6, CRP, ferritin, and cytokeratin-18, have been proposed as useful predictors of NAFLD/NASH in the past, but none of them have shown sufficient sensitivity and specificity in clinical routine [33]. Campos et al. developed a clinical scoring system for predicting NASH in morbidly obese patients [16]. In our cohort, liver function capacity is correlated to the NASH clinical scoring system. We decided to use this scoring system as it is based on data from obese patients who underwent bariatric surgery. Furthermore, the parameters for the NASH clinical scoring system can easily be obtained in standard evaluation of obese patients in a bariatric center. However, this clinical scoring system is not established in clinical routine, and ethnicity plays a minor role in a regular German bariatric surgery cohort.

Liver fibrosis is an important determinant of long-term outcomes in NAFLD and a robust predictor of liver-related mortality [34, 35]. Buechter et al. showed a strong correlation between LiMAx values and fibrosis in chronic liver diseases [36].

The LiMAx® test is not suitable for smokers (>15 cigarettes per day). Acute cigarette smoking interferes with ^13^C-methacetin breath tests and regular smoking induces cytochrome P450 1A2 activity [37, 38]. However, since patients are advised to be fasting, the influence of acute cigarette smoking in clinical practice is limited [39].

## 5. Conclusions

This study provides the first comparison of liver biopsy to the LiMAx® test in more than 100 obese patients. The liver function capacity correlates with histological features and clinical scoring systems. LiMAx® testing as a noninvasive tool is able to distinguish definite NASH from not NASH in morbidly obese patients. This could facilitate screening of obese individuals and other suspect cases for NASH. Furthermore, repetitive LiMAx® testing might enable monitoring of disease progression and evaluation of the response to therapeutic interventions, including bariatric surgery.

## Figures and Tables

**Table 1:** Patients' characteristics.

*Demographic data*	*n*	
Male	32	31.3%
Female	70	68.6%
Age (years)	43.2	±10.5
BMI (kg/m^2^)	53.7	±9.3
*Comorbidities*		
Type 2 diabetes mellitus	37	36.2%
Hypertension	65	63.7%
Obstructive sleep apnea syndrome	50	49.0%
*Surgery*		
Roux-en-Y gastric bypass	43	42.2%
Sleeve gastrectomy	59	57.8%
*NAFLD activity score*		
No NASH	35	34.3%
Borderline	40	39.2%
Definite NASH	27	26.5%
Median NAS	3	IQR = 3
*NASH risk classification*		
Low	14	13.7%
Intermediate	32	31.4%
High	26	25.5%
Very high	30	29.4%

## Data Availability

The data used to support the findings of this study are available from the corresponding author upon request.

## Abbreviations

ALT: Alanine aminotransferase
AST: Aspartate aminotransferase
AUROC: Area under receiver operating characteristic
BMI: Body mass index
ICG: Indocyanine green test
IQR: Interquartile range
LiMAx®: Liver maximum capacity test
NAFLD: Nonalcoholic fatty liver disease
NAS: NAFLD activity score
NASH: Nonalcoholic steatohepatitis
T2DM: Type 2 diabetes mellitus.
